# Facile fabrication of porous Bi_x_S_y_/Si photodetectors by one step laser ablation in liquid

**DOI:** 10.1038/s41598-026-37668-8

**Published:** 2026-02-10

**Authors:** Ahmed M. Ahmed, Asmiet Ramizy, Raid A. Ismail, Ethar Yahya Salih, O. Aldaghri, M. H. Eisa

**Affiliations:** 1 Department of Physics, College of Science, University of Anbar, Anbar, Iraq; 2 College of Applied Science, University of Technology, Baghdad, Iraq; 3 College of Energy and Environmental Sciences, Al-Karkh University of Science, Baghdad, 10081 Iraq; 4 Department of Physics, College of Science, Imam Mohammad Ibn Saud Islamic University (IMSIU), Riyadh, 13318 Saudi Arabia

**Keywords:** Bismuth sulfide, Bi_x_S_y_/Si photodetector, Laser ablation, Thiourea, Laser fluence, Materials science, Nanoscience and technology, Optics and photonics, Physics

## Abstract

In this study, facile bismuth sulfide (Bi_x_S_y_) porous nanostructures (NSs) were synthesized by the liquid-based pulsed laser ablation (PLAL) method. The Bi_x_S_y_ nanostructures were formed by laser ablation of the bismuth (Bi) target within 1 M of thiourea [SC (NH_2_)_2_] aqueous solution, along a fixed 400 laser pulses. The influence of laser fluence on the microstructural, morphological, chemical, optical, and electrical features of Bi_x_S_y_ porous film has been systematically examined. X-ray diffraction (XRD) analysis confirmed that all synthesized Bi_x_S_y_ nanoparticles (NPs) demonstrated a poly-crystalline nature along orthorhombic crystal orientation. As the fluence of laser amplified from 6.37 J/cm²/pulse to 15.92 J/cm²/pulse, the attained direct bandgap of Bi_x_S_y_ reduced from 1.8 to 1.69 eV. Photoluminescence (PL) measurements showed a single emission peak at 672, 677, 712, and 729 nm for Bi_x_S_y_ NSs prepared at 6.37, 9.55, 12.74, and 15.92 J/cm²/pulse, respectively. Raman spectroscopy revealed three vibrational modes positioned around 294, 510, and 654 cm⁻¹. Field emission scanning electron microscope (FE-SEM) images displayed the construction of a porous, grid-like nanostructure, with particle sizes ranging from 34.08 nm to 44.65 nm for sample fabricated with 15.92 J/cm²/pulse fluence. The dark I-V features of Al/n-Bi_x_S_y_/p-Si/Ag heterostructured photodetectors demonstrated rectifying behavior. Under incident light, the photo current-voltage (I-V) properties indicated high photosensitivity. A device attained at fluence of 12.74 J/cm²/pulse exhibited the highest responsivity (1.139 A/W) and specific detectivity (1.68 × 10^¹³^ Jones) at 375 nm. Additionally, the highest external quantum efficiency (EQE) of 376.52% was achieved at 375 nm for the same photodetector. The time-resolved analysis (ON/OFF states) are also demonstrated by means of laser fluence.

## Introduction

Bismuth sulfide (Bi_2_S_3_), commonly known as bismuthinite, is the most prevalent natural form of bismuth and belongs to the family of chalcogenide semiconductor materials due to its chalcogenide nanostructure^1^. Bi_2_S_3_ is a generally non-toxic n-type semiconductor with a number of unique characteristics, including high electrical conductivity, a strong absorbance coefficient (ranging from 10^4^ to 10^5^ cm⁻¹), and a significant conversion rate of incident photon to electron (~ 5%). Its direct bandgap energy, which ranges from 1.30 to 1.70 eV in bulk, is well-suited for photovoltaic and photodetector applications. Variations in the bandgap are often attributed to changes in stoichiometry or quantum confinement effects due to size reduction^2–4^.

Bi_2_S_3_ has a unique lamellar-like coordination structure composed of Bi^3+^ and S^2-^ ions arranged in infinite chains^3^. Upon crystallization, it adopts an orthorhombic crystal system^3,5,6^. These properties, along with its non-toxicity, make Bi_2_S_3_ a desirable material for applications such as sensors^7^, energy storage^8^, photocatalysis^9^, and photovoltaics^10^. Recent studies have highlighted its structural flexibility, enabling the synthesis of diverse morphologies, including nanoparticles, nanorods, nanowires, nanofabrics, and microstructures, depending on the synthesis method used^11,12^. Bi_2_S_3_ nanoparticles (NPs) and thin films (TFs) have been successfully synthesized using variety of chemical and physical approaches such as chemical vapor deposition (CVD)^1^, rapid thermal evaporation techniques (RTET)^10^, ultrasonic methods^13^, chemical bath deposition (CBD)^14^, pulsed laser deposition (PLD)^15^, and colloidal methods^16^. Among these, the pulsed laser ablation in liquid phase (PLAL) technique has emerged as a highly innovative and effective approach for controlling particle size and producing nanostructured materials^17^.

PLAL relies on highly intense ablation of pulsed laser to create an interaction between laser and target’s surface, generating a plasma plume entirely submerged in liquid. This process enables the synthesis of nanostructured materials in various shapes and sizes. Compared to other methods, PLAL is considered a highly adaptable, cost-effective, and straightforward technique for producing high-purity materials, including pure or hybrid metal nanoparticles^18,19^. Sun and Tsuji^20^ prepared stoichiometric Sb_2_S_3_ nanoparticles using water-based laser ablation with H_2_S solution. A pulsed laser ablation of CdS in liquid medium was established to prepare tunable size (5–12 nm) and diverse shapes such as rope, rod, sphere, etc^21^. Bi_2_S_3_ nanoparticles were attained utilizing pulsed laser ablation method via ablation of bismuth pellet in solution of H_2_O_2_ with laser radiation (355 nm)^22^. A crystalline bismuth-based metal organic framework was synthesized via ablation of pulsed laser in liquid environment as bottom-up technique^23^. In optoelectronic applications, Shkir, M., & Alshahrani, T. fabricated Bi_2_S_3_:Nd photodetector by spray pyrolysis with responsivity (R), external quantum efficiency (EQE), detectivity (D*), and rapid rise/fall time of 1.36 × 10^− 1^ A/W, 43%,3.05 × 10^10^ Jones, and 0.1/0.2 s., respectively, for Bi_2_S_3_:Nd 3 wt% thin film photodetector^24^. Cheng, Jiahao, et al. presented a new 2D LA-Bi_2_S_3_ van der Waals photodetectors prepared by a spin-coating thermal annealing method exhibit detectivities of 1.69 × 10^12^ and 2.6 × 109 cm·Hz^1/2^ W^− 1^ at 638 nm and 1310 nm illuminations, respectively and responsivities of up to 3.6 mA/W^25^. Kumar, S. Sathish, et al., utilized nebulizer spray pyrolysis approach for Bi_2_S_3_ thin film deposition considering different temperature (250–375 °C); the attained photo-responsive characteristics demonstrated values of 0.16 A/W, 9.75 × 10^9^Jones, 50.8% and rise/fall time of 0.3/0.4 sec^26^.

To the best of our knowledge, no prior data has reported the synthesis of Bi_2_S_3_ via laser ablation of a bismuth target in a thiourea solution. In this work, we propose a novel and rapid method for synthesizing colloidal Bi_2_S_3_ porous nanostructures (NSs) via the ablation of bismuth in thiourea solution using 532 nm wavelength Nd: YAG laser at various laser fluences. This pathway is catalyst-free and offers a promising route for fabricating high-purity Bi_2_S_3_ NSs. During the process of thermal decomposition in thiourea medium along laser irradiation, a reactive ablation process leads to single-step and direct formation of pure-phase/crystalline Bi_x_S_y_ without post-thermal treatment needed. As such, a porous nanostructure is attained during this unique pathway. This in turn allows highly effective wavelength absorption for optoelectronic application. A range of techniques, including X–ray diffraction (XRD, atomic force microscope (AFM), field-emission scanning electron microscope (FESEM), Raman spectroscopy, UV-Vis spectroscopy, and photoluminescence, were employed to thoroughly characterize the colloidal Bi_2_S_3_ porous NSs. For the first time, a hybrid nanostructured Al/n- Bi_2_S_3_/p-Si/Ag heterojunction (HJ) photodetector (PD) was proposed. Additionally, a detailed examination of the laser fluence effect on the performance of n- Bi_2_S_3_/p-Si photodetectors was conducted. Figures-of-merit of the demonstrated devices, including I-V behavior, R, EQE, D*, and response time, were comprehensively analyzed and analyzed.

## Experimental

**Fig. 1:**
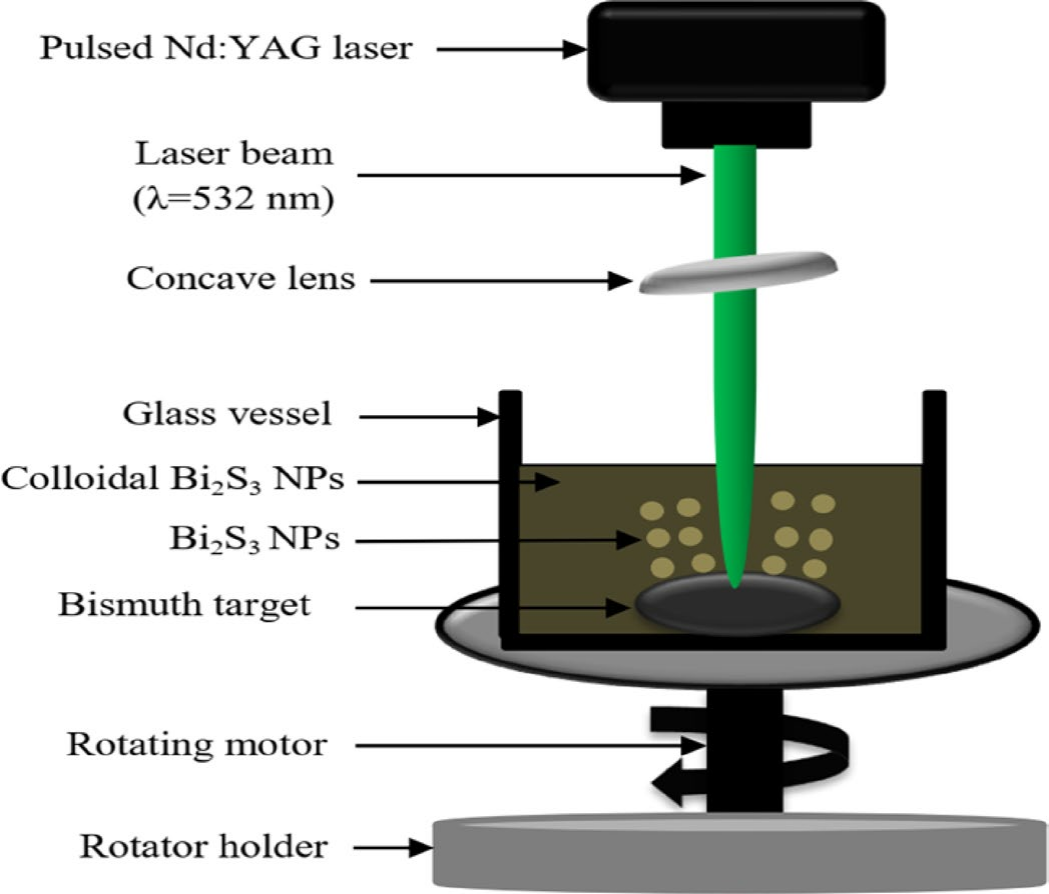
Schematic diagram of PLAL system.

Bi_x_S_y_ porous nanostructures (NCs) were prepared via pulsed laser ablation of a high-purity bismuth target in a 1 M thiourea [SC(NH_2_)_2_] aqueous solution. The solution was prepared by dissolving 0.228 g of thiourea pellets (molecular weight 76.12 g/mol) in 3 ml of distilled water at room temperature. The weight of the all chemical was measured using 4-digit balance (Sartorius). To create bismuth pellets, 5 g of high-purity (99.88%) bismuth fine powder (purchased from Sigma-Aldrich) was compressed using a hydraulic press (MEGA) under 10 tons, forming disks with a 2 cm diameter and 0.3 cm thickness. The laser beam from a Q-switched Nd: YAG laser with λ = 532 nm, pulse duration of 7 ns, TEM_00_ mode, repetition frequency of 6 Hz was focused vertically onto the bismuth target placed at the bottom of a glass vessel and the height of thiourea solution was 5 mm above the target. Ablation was performed at a fixed number of 400 laser pulses with fluences of 6.37, 9.55, 12.74, and 15.92 J/cm²/pulse. The laser spot size, measured using an optical microscope, was approximately 1 mm on the bismuth target. Figure 1 shows the schematic diagram of laser ablation of Bi_2_S_3_ NSs in liquid system. The laser beam was focused on the target using a converging lens (convex) with focal length of 7 cm. The vessel was rotated during laser ablation with rotating speed of 300 rpm using DC steeper motor.

**Fig. 2:**
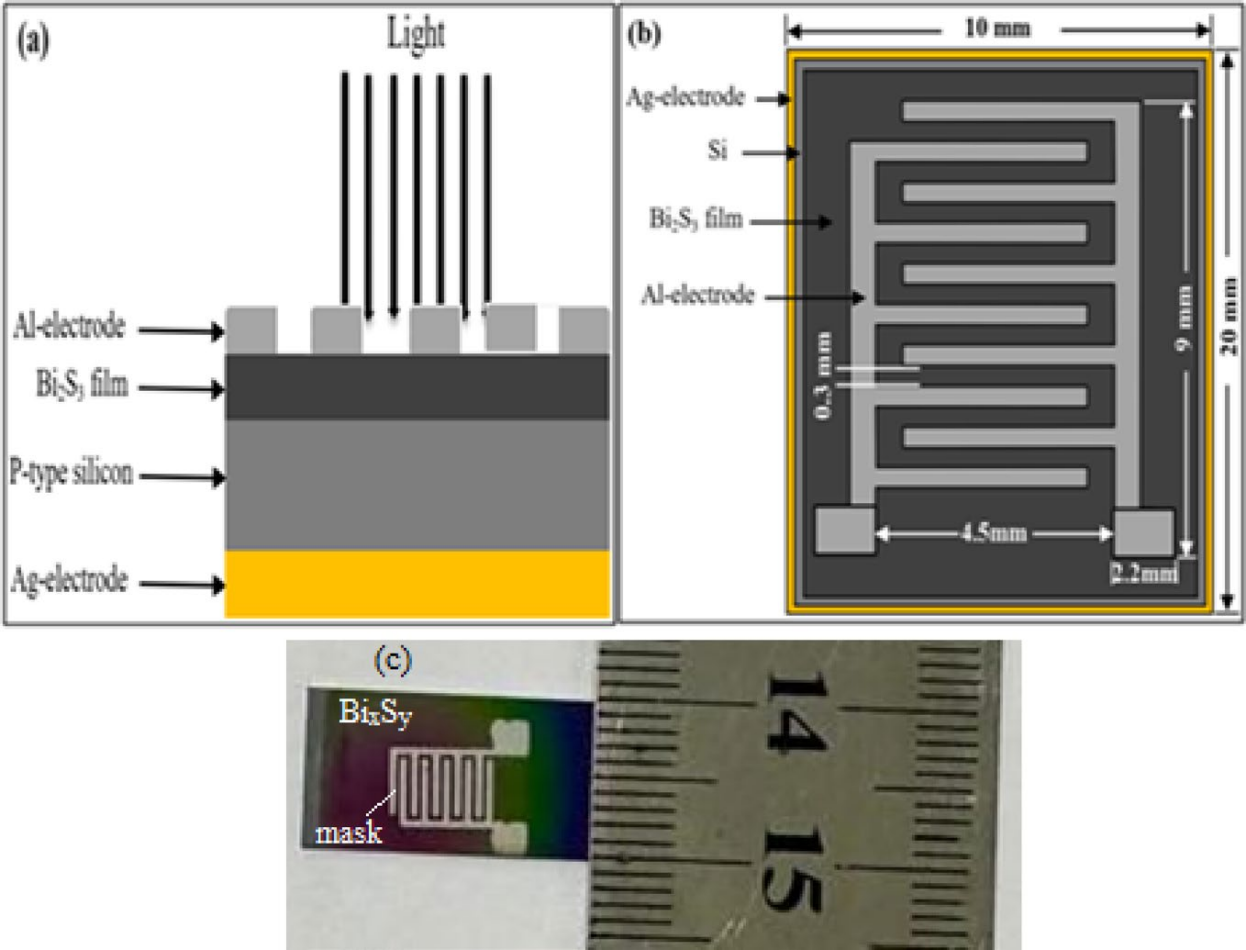
**(a)** Schematic diagram of cross-sectional view diagram of Bi_x_S_y_/p-Si photodetector, **(b)** photograph of the photodetector’s interdigit electrode dimensions, and **(c)** optical image of the photodetector.

Bi_x_S_y_ porous films were deposited onto silicon substrates using a spin-coating method at 1500 rpm for 3 min. Structural characterization was performed using an X-ray diffractometer (Shimadzu XRD-6100). Optical absorption spectra and photoluminescence were measured using a UV-Visible dual-beam spectrophotometer (T80 UV/VIS) and a Cary Eclipse spectrometer, respectively. Raman spectra of Bi_x_S_y_ NPs were acquired using a Bruker Senterra Raman microscope (532 nm). Morphological analysis was carried out using field emission scanning electron microscopy (FESEM, Inspect F50) and atomic force microscopy (AFM, Angstrom Advanced SPM AA3000). Al/n-Bi_2_S_3_/p-Si/Ag heterojunction photodetectors were fabricated by depositing Bi_x_S_y_ colloidal NS film with thickness of 200 nm onto p-type mirror-like silicon substrate (dimensions: 2 × 1 cm², thickness: 525 μm, and resistivity: 1–10 Ω.cm) using spin coating technique. Prior to deposition, the silicon wafers were cleaned with a 1:10 solution of HF and H_2_O to eliminate the layer of native oxide, immersed in alcohol for 10 min, and rinsed with deionized water. Ohmic contact was recognized through thermally evaporated aluminum film onto the Bi_2_S_3_ layer and a silver film onto the back of the silicon substrate using an interdigitated mask. A schematic cross-sectional view of the photodetector is shown in Fig. 2(a), the interdigitated mask dimensions are illustrated in Fig. 2b, and optical image in Fig. 2c.

Current–voltage (I–V) characteristics of the Al/n- Bi_x_S_y_/p-Si/Ag photodetectors were measured at room temperature using a photodetector evaluation system comprising a digital DC power supply, a UNI-T UT33C digital electrometer, a halogen lamp, a monochromator, and a silicon power meter. The stability of the photodetector was conducted via measured the rise and fall times at 300 K and humidity of 45%.

## Results and discussion

**Fig. 3:**
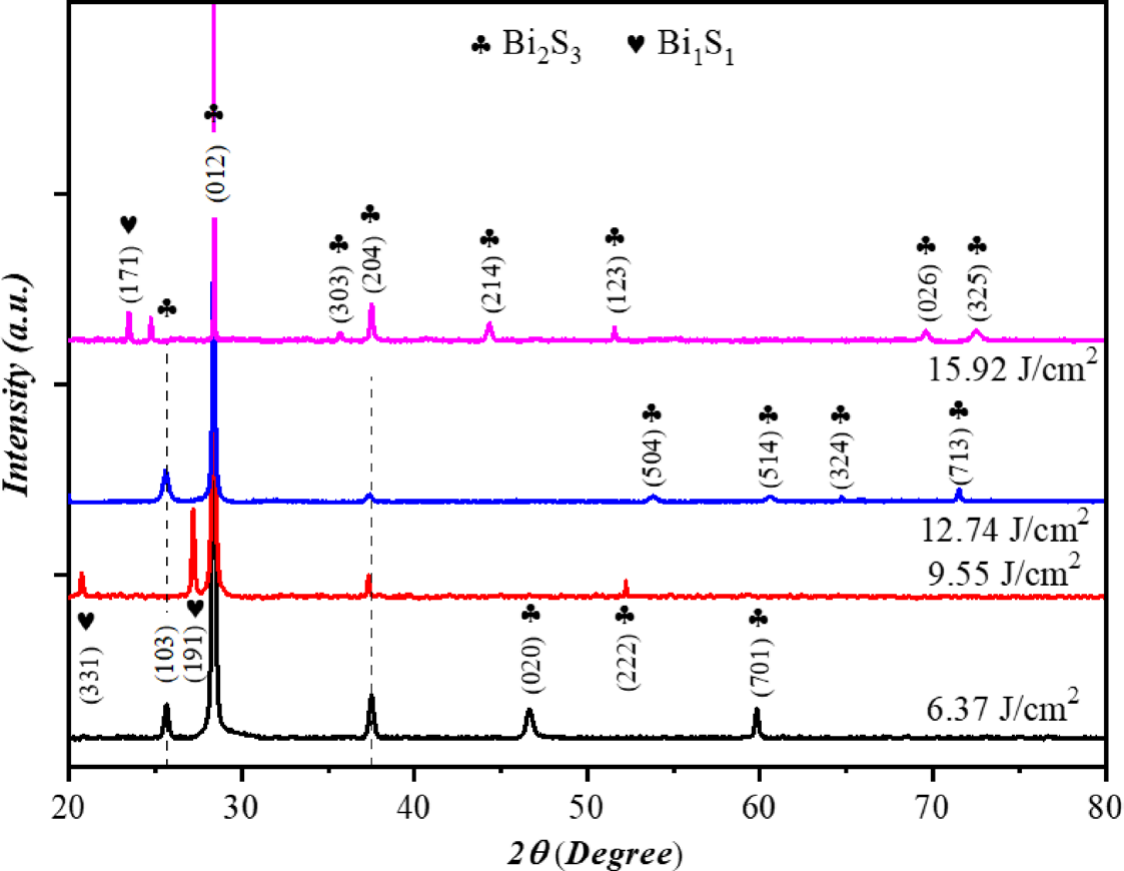
XRD patterns of Bi_x_S_y_ NS produced at various laser fluences.

Figure 3 depicts the XRD pattern of Bi_x_S_y_ porous NSs prepared at various laser fluences. The XRD pattern of Bi_x_S_y_ prepared at 6.37 J/cm²/pulse laser fluence showed the presence of five peaks observed at 2θ = 25.6°, 28.3°, 37.5°, 46.6°, and 59.8°, which correspond to (103), (012), (204), (020), and (701) planes, respectively. The observed XRD peaks of Bi_x_S_y_ for all laser fluences are indexed to polycrystalline with an orthorhombic structure of Bi_x_S_y_ according to ICSD #98-015-3948, ICSD #98-015-3950, ICSD #98-017-1864^27^ and three peaks belong to BiS according to ICSD #98-007-9515. As clearly seen in Fig. 3, the highest peak intensity was shown in the direction of the (012) plane, suggesting that the deposited particles tend to orient preferentially or aggregate in a direction normal to the substrate surface, influenced by their morphology and the kinetic energy imparted during synthesis at higher laser fluences. A slight shift in 2θ of the reflection peaks occurred at higher laser fluences from 9.55 to 15.92 J/cm^2^/pulse, which may suggest the presence of internal stress induced by the rapid formation and quenching of nanoparticles at high laser fluence, as inferred from changes in XRD peak profiles. The crystallite size (D) of Bi_x_S_y_ films prepared at various laser fluences was calculated with the aid of Scherrer’s formula, as shown in Eq. (1)^28^. The interplanar distance (d) was computed utilizing the formula as presented in Eq. (2)

1 $$\:{D}_{ave}=\:\frac{K\lambda\:}{\beta\:\:cos\theta\:}\:$$

2 $$\:D=\frac{n\lambda\:}{2\:sin\theta\:}\:$$

where K = 0.9, λ is the wavelength of X-ray of Cukα source (0.1405 nm), β is the full width at half maximum, and $\:\theta\:$ is the diffraction angle. The crystallite size of Bi_x_S_y_ NSs films along (012) plane synthesized as a function of laser fluence is shown in Table 1. The crystallite size was found to be increased as the laser fluence increases. This result can be attributed to the increased energy delivered to the Bi target, which causes greater material ablation and the formation of a denser plasma plume. On the other hand, increases the laser fluence results in decreases the cooling rate and leads to better lattice ordering and hence high crystallinity. The dense environment promotes particle coalescence and growth within the cavitation bubble. The dislocation density (δ) and lattice strain (ε) generated in the prepared films that coincided with the laser ablation operation were computed utilizing the formulas as presented below.

3 $$\:\delta\:\:=\frac{1}{{D}^{2}}$$

4 $$\:\varepsilon\:=\:\frac{\beta\:\:cos\theta\:}{4}$$

As shown in Table 1, the sample prepared with 15.92 J/cm^2^ exhibits lowest dislocation and lattice strain due to the fact that high laser fluence leads to formation of high temperature and this can be annealed the structural defects. The nanoparticles synthesized with lower laser fluence have rapid cooling rate (quenching) and the will produces structural defects and internal stresses.

**Table 1:** XRD analysis of the Bi_x_S_y_ nanostructure films prepared at various laser fluences along (012).

Laser fluence (J/cm^2^/pulse)	2$\:\boldsymbol{\theta\:}$ (Deg.)	FWHM (Deg.)	D (nm)	d (nm)	δ (nm)^−2^ 10^− 4^	ε% 10^− 5^
6.37	28.37	0.23	29	0.314	7.89	97
9.55	28.37	0.27	35	0.314	11.49	118
12.74	28.38	0.12	69	0.314	2.09	50
15.92	28.39	0.11	78	0.314	1.61	44

**Fig. 4:**
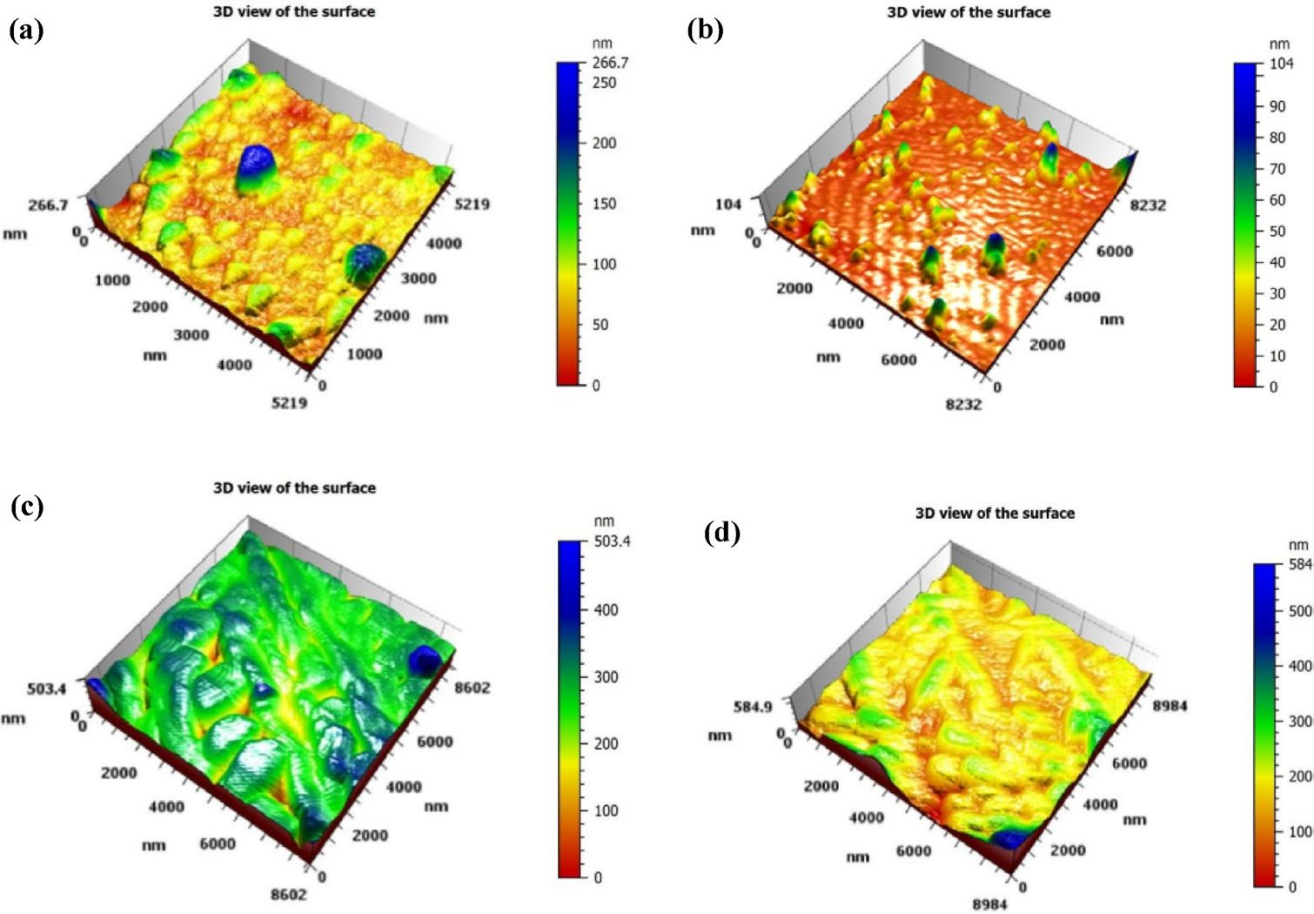
3D AFM images of Bi_x_S_y_ NSs attained at **(a)** 6.37, **(b)** 9.55, **(c)** 12.74, and **(d)** 15.92 J/cm^2^/pulse.

Figure 4 demonstrates the 3D AFM images of the prepared nanostructured films which gives information about the surface topography and surface roughness of Bi_x_S_y_ NSs film. The 3D AFM image of the Bi_x_S_y ​_ porous nanostructures (NSs) shows a rough and porous surface topography, characterized by irregular peaks and valleys. Increasing the laser fluence to 9.55 J/cm^2^, as shown in Fig. 4(b), results in a smoother surface compared to that in Fig. 4(a), with lower peak heights and more uniform features. As shown in Table 2, the root mean square (RMS) of the surface roughness decreases. This surface appears more homogeneous, with fewer sharp peaks and valleys. This could be due to optimized laser fluence during synthesis, which reduces surface irregularities. We suggest that some porous structures formed during the initial laser pulses are subsequently ablated by continued laser irradiation, and the resulting ablated material may redeposit locally on the surface, partially filling voids and smoothing the morphology. However, further increases in laser fluence result in an increase in the surface roughness of the films, which can be attributed to the formation of larger or more irregular nanoparticles due to stronger shockwaves and cavitation dynamics during ablation. These particle-level effects influence the morphology of the deposited film, including its roughness.

**Table 2:** RMS surface roughness and grain size-based fluence of laser attained using AFM investigation.

Laser fluence (J/cm^2^/pulse)	Grain Size (nm)	RMS surface roughness (nm)
6.37	85	47
9.55	64	34
12.74	167	53.74
15.92	472	99.70

**Fig. 5:**
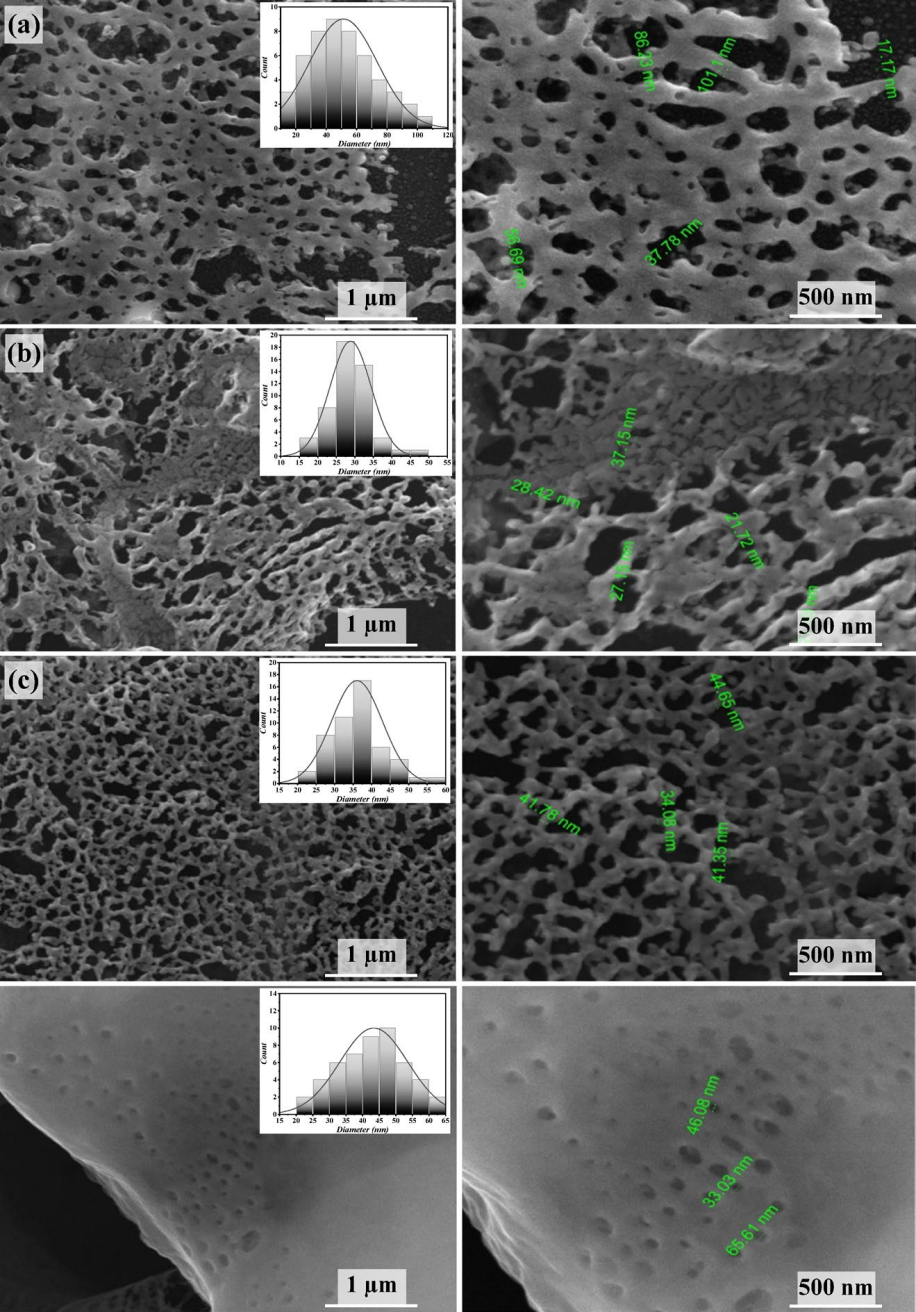
FE-SEM of Bi_x_S_y_ NPs (left − 1 μm) and magnified FE-SEM (right – 500 nm) prepared at various laser fluences: **(a)** 6.37, **(b)** 9.55, **(c)** 12.74, and **(d)** 15.92 J/cm^2^/pulse, with 400 pulses.

Figure 5 reveals the FE-SEM images of Bi_x_S_y_ Bi_2_S_3_ NSs arranged at several fluences of laser. In Fig. 5 (a-b), the FE-SEM image of Bi_x_S_y_ films obtained with 6.37 and 9.55 J/cm^2^/pulse, demonstrate the formation of matrix of porous morphology. As observed, the pore size of the films prepared at 6.37 and 9.55 J/cm^2^/pulse varied from 17.17 to 101.1 nm and from 21.72 to 37.15 nm, respectively. The Bi_2_S_3_ film synthesized at 12.74 J/cm^2^/pulse, as illustrated in Fig. 5 (c), exhibited the forming of high density of pores 3D network porous grid-like structure. Figure 5 (c) depicts a highly porous material with interconnected voids and channels, featuring pore sizes ranging from approximately 34 nm to 44 nm and thin walls separating the pores.

**Fig. 6:**
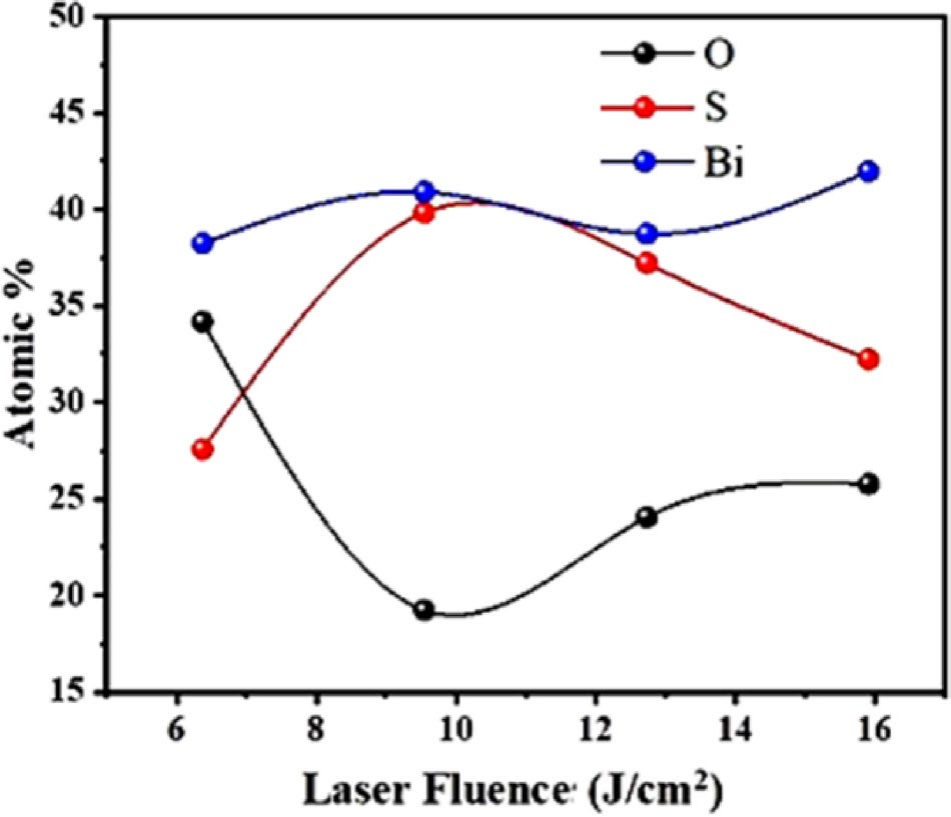
Variation of atomic percentage of Bi, S, and O elements of Bi_x_S_y_ films with laser fluence.

The analysis indicates that the porous surface area occupies approximately 78.98% of the total area in the FE-SEM image. This high porosity suggests a significant amount of void space within the material, which can enhance surface interactions and is beneficial for applications such as photodetection. The uniform distribution of pores suggests a well-controlled fabrication process, resulting in a consistent structure. This porous network offers a high surface area, making it suitable for applications such as photodetection. This morphology enhanced light trapping. However, the thin walls may pose challenges for mechanical stability and scaling up the fabrication process. The Bi_x_S_y_ film performed at 15.92 J/cm^2^/pulse, reveals the formation porous sponge-like structure with various size ranges from 33.03 to 65.61 nm, as presented in Fig. 5(d). The variation of atomic percentage of Bi, S, and O elements of Bi_x_S_y_ films with laser fluence is presented in Fig. 6. Although oxygen was perceived during EDX analysis, there is no peak corresponded to the formation of Bi_2_O_3_ layer in the XRD patterns; this in turn suggests that Bi_2_O_3_, detected in the EDX spectra is either amorphous or a very thin layer.

The EDX spectra confirm the existence of bismuth, sulfur, and oxygen elements which are the main constitutes of the Bi_x_S_y_ film. No other elements were uncovered in these spectra, which confirm the high purity of the film. The stoichiometry of the Bi_x_S_y_ film was evaluated from the [Bi]/[S] atomic ratio, obtained from EDX analysis. Actually, the Bi_x_S_y_ film has a stoichiometric Bi: S ratio of 2:3 (1.5). Bi/S atomic ratios were estimated from EDX analysis for Bi_2_S_3_ films fabricated at laser fluences of 6.37, 9.55, 12.74, and 15.92 J/cm²/pulse, and were found to be approximately 1.05, 1.02, 1.24, and 1.37, respectively. The films prepared at 6.37, 9.55, and 12.74 J/cm^2^/pulse appear slightly off-stoichiometric, while the one at 15.92 J/cm^2^/pulse approaches the ideal Bi_x_S_y_ composition. However, EDX cannot distinguish between Bi bonded to sulfur and Bi bonded to oxygen. Given the high oxygen content detected in all samples, it is highly likely that a portion of Bi is bonded to oxygen, forming Bi-O bonds, contributing to the elevated Bi content and underestimating sulfur in the calculated Bi/S ratios. This effect should be considered when interpreting compositional data. The formation of this phase influences the Bi/S ratio obtained from EDX by altering the film’s overall stoichiometry. This sample’s apparent deviation from the ideal Bi_x_S_y_ stoichiometry is due to both measurement limitations and phase heterogeneity within the material.

**Fig. 7:**
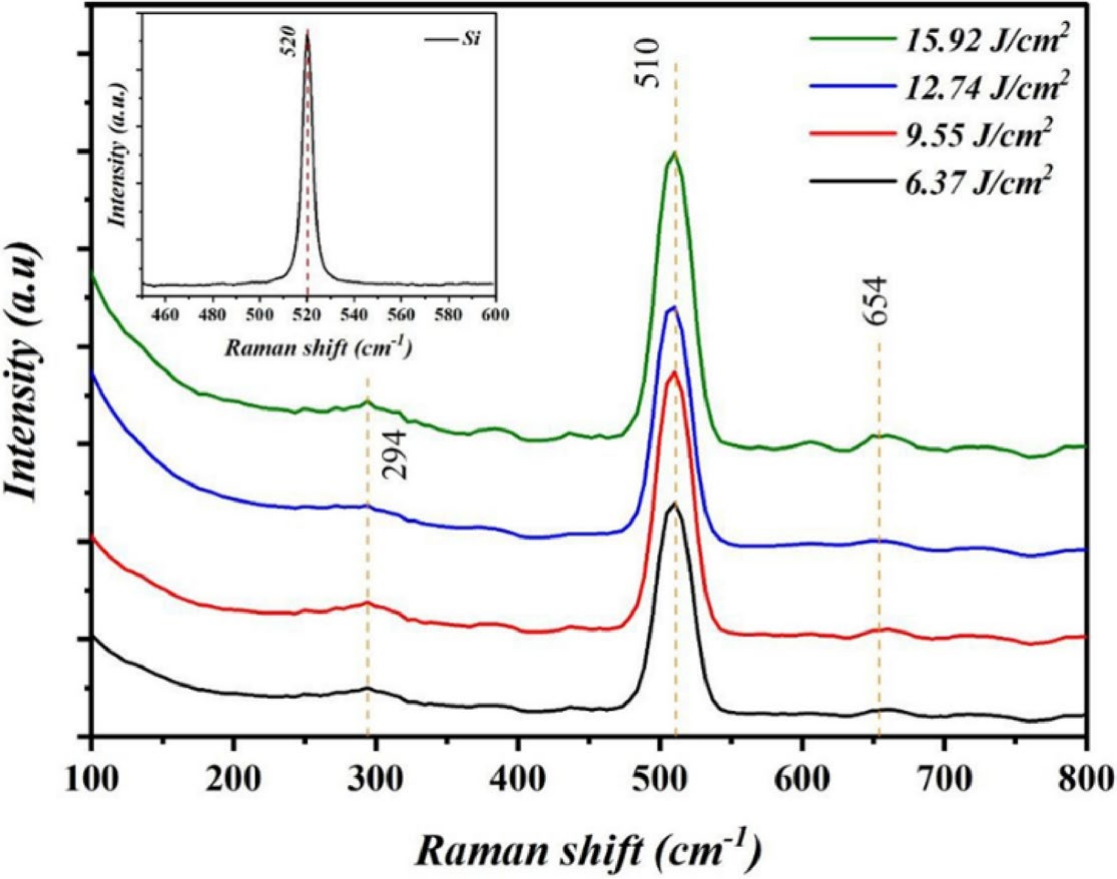
Raman spectra of colloidal Bi_x_S_y_ porous film deposited on silicon prepared at various laser fluences. Inset is the Raman spectrum of silicon substrate.

Figure 7 illustrates the effect of laser fluence on Bi_x_S_y_ nanostructured film’s Raman spectra of. The spectra exhibit Raman peaks in 100–800 cm^− 1^ range of, corresponding to the crystalline bismuth sulfide phase. Three distinct vibrational modes are observed in the Raman spectra of Bi_x_S_y_ porous NSs. Two Raman peaks, located at approximately 294 cm^− 1^ and 654 cm^− 1^, are related to the A_g_ mode, while a third peak at 510 cm^− 1^ corresponds to the Bi-S bond stretching ​mode^29–31^. The B_1g_ and A_g_ phonon modes arise, respectively, from the longitudinal and transverse vibrations of sulfur (S) atoms^32^. As the laser fluence increases, the intensity of the Raman peaks growths, and their FWHM declines. These changes can be attributed to improved film crystallinity, an increase in the concentration of Bi_x_S_y_ nanostructures, and an increase in the grain size of the film. The Bi_x_S_y_ nanostructured film show weak Raman peaks at 294 cm^−1^ and 654 cm^−1^, indicating low intensity vibrational modes that are less Raman active than the dominant Ag and B_1_g modes. Their low intensities are further reduced by nanoparticle size effects like phonon confinement and surface disorder, which reduced higher-frequency vibrations. The inset of Fig. 7 is the Raman spectrum of silicon substrate, which confirms the presence of a single Raman peak at 520 cm^−1^.

**Fig. 8:**
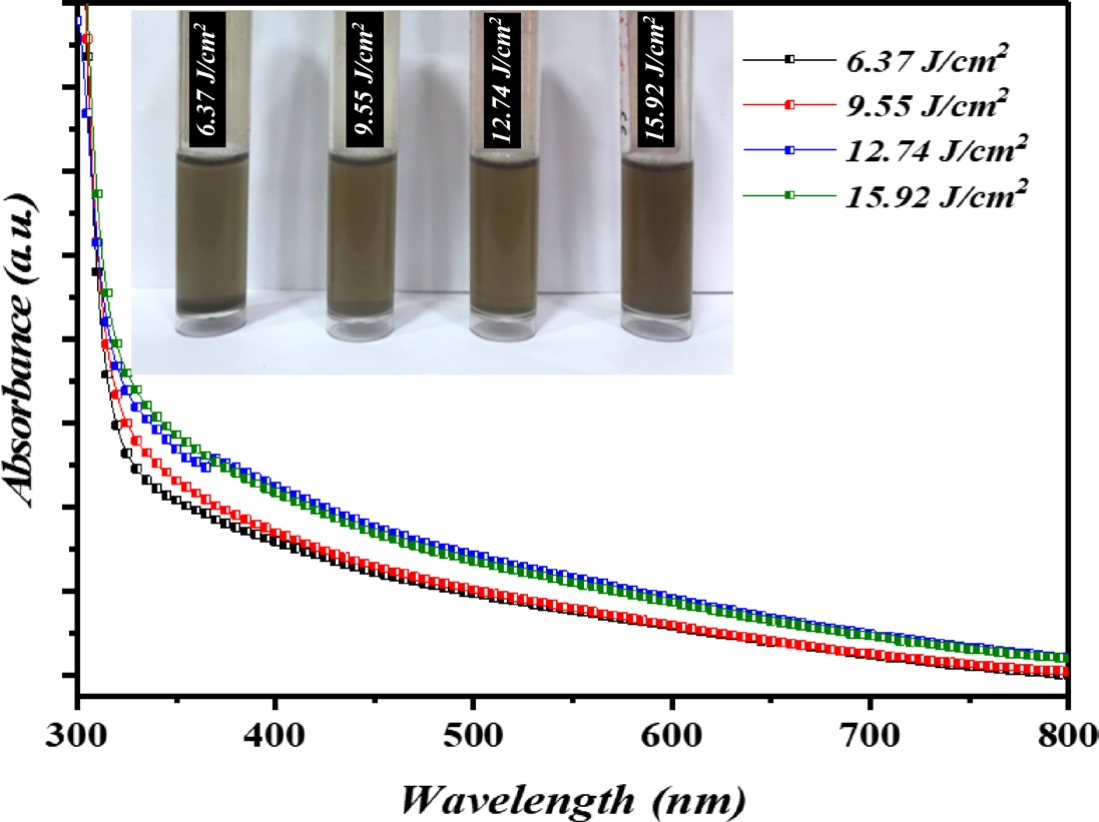
Optical absorption of Bi_x_S_y_ colloid synthesized at various laser fluences for 400 pulses.

**Fig. 9:**
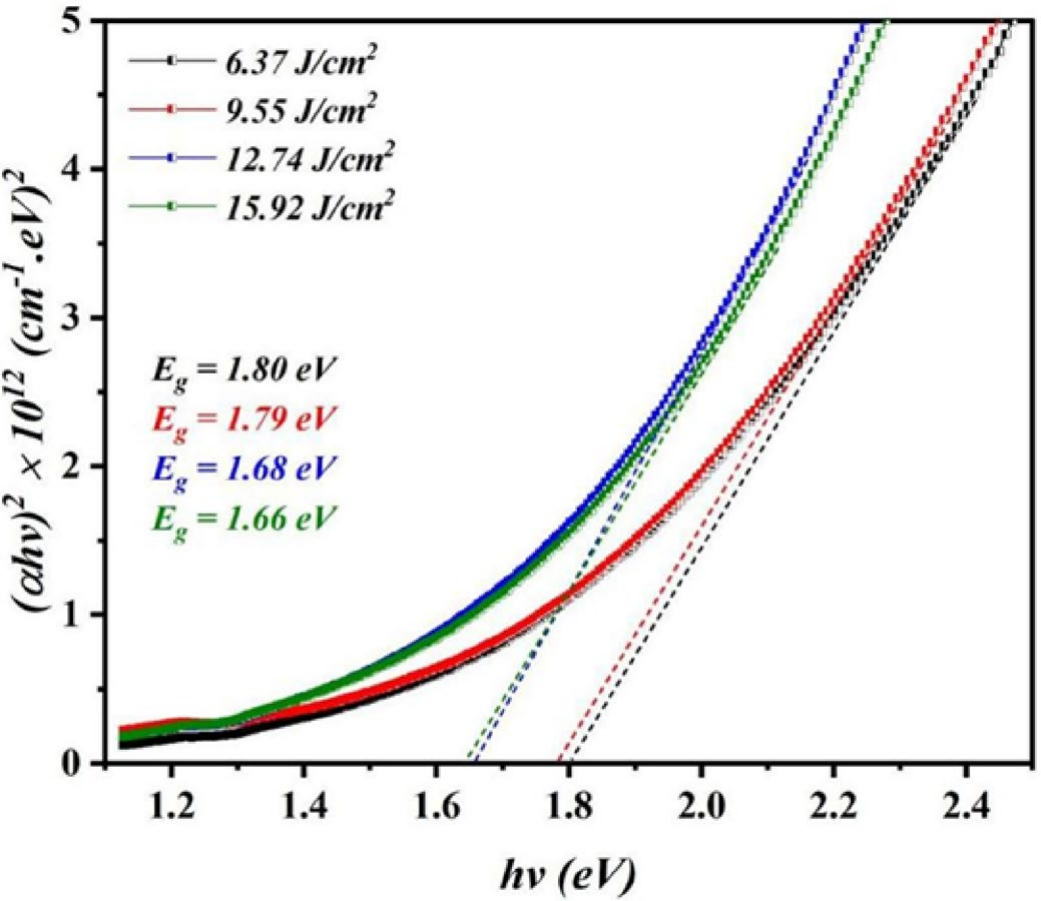
Variation of $\:{\left(\alpha\:h\nu\:\right)}^{2}$ against photon energy ($\:h\nu\:$) of Bi_x_S_y_ colloid synthesized at various laser fluences for 400 pulses.

Figure 8 shows the absorption of colloidal Bi_x_S_y_ porous at various laser fluences. The optical absorption of Bi_x_​S_y_ was found to be increased as the laser fluence increases due to an increase in both concentration and size of the film. The UV-Vis absorption spectrum of the Bi_x_S_y_ film synthesized at 6.37 J/cm²/pulse reveals a sharp absorption edge near 315 nm, indicating the onset of strong absorption caused by interband electronic transitions. Beyond this point, absorption gradually decreases because the incident photon energy is insufficient to excite electrons across the bandgap. The extended absorption tail up to ~ 800 nm could be due to sub-bandgap absorption caused by defects or impurity states. The energy gap of the bulk Bi_x_S_y_ is typically in the range of 1.3–1.5 eV (950–825 nm). The blue-shift in the absorption edge compared to bulk Bi_2_S_3_ indicates that structural disorder, surface states, or quantum confinement effects could impact the optical properties of the thin film. The inset of Fig. 8 illustrates the photographs of colloidal Bi_x_S_y_ porous NSs synthesized at various laser fluences. As clearly seen, colloidal Bi_x_S_y_ NPs exhibit a noticeable color change from light brown to dark brown with an increase in laser fluences, which can be attributed to a higher concentration of ablated nanoparticles as well as change in particle size of Bi_2_​S_3_. The direct optical bandgap of Bi_x_S_y_ NPs was calculated using Tauc’s relationship:

5 $$\:{\left(\alpha\:h\nu\:\right)}^{2}\:=\:A\:\left(h\nu\:\:-\:Eg\right)$$

where *A* is the constant depends on the material type, α is the absorption coefficient, and *hv* is the photon energy^33,34^. Figure 9 depicts the variation of photon energy ($\:h\nu\:$) and $\:{\left(\alpha\:h\nu\:\right)}^{2}$ and the direct band gap energy can be found by the extrapolating the line upon the photon energy ($\:h\nu\:$) axis. The findings obtained verified that as the laser fluence rose from 6.37 J/cm^2^/pulse to 15.92 J/cm^2^/pulse, the direct optical energy gap energy of Bi_x_S_y_ ranged from 1.8 to 1.66 eV. The expected uncertainty in the presented bandgaps to be ± 0.1 eV based on the variability within the linear fitting region.

This decrease in the value of energy gap is due to the increasing the particle size with laser fluence, which is in agreement with FE-SEM study. The resulting energy band gap has a value greater than that of bulk bismuth sulfide as a result of quantum size effect (QSE)^35^. Furthermore, the variation of optical energy gap can be also ascribed to film stoichiometric^36^. The quantum confinement leads to increase the kinetic energy of charge carriers and consequently raising the effective energy gap of the Bi_x_S_y_. On the other hand, the broad PL peak can be attributed to the presence of different confinement strengths and particle sizes.

**Fig. 10:**
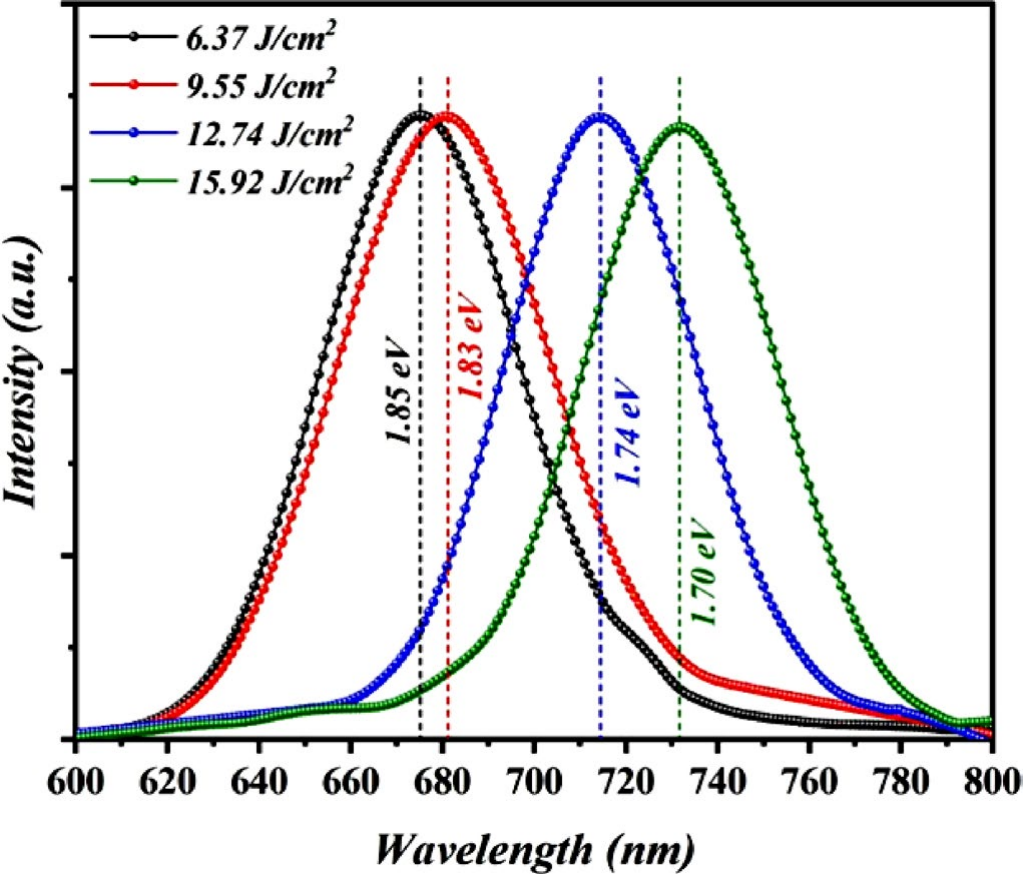
Photoluminescence spectra of Bi_x_S_y_ NSs prepared with various laser fluences.

Figure 10 displays the Photoluminescence spectra of porous Bi_x_S_y_ nanostructure films fabricated at several laser fluences animated by a 320 nm laser beam at room temperature. The sample synthesized at 6.37 J/cm^2^/pulse displays PL peak emission situated at 672 nm, which corresponds to 1.85 eV. This is associated with radiative recombination involving electron–hole pairs at the extrinsic impurity sites. The photoluminescence of the Bi_x_S_y_ film grown with 9.55 J/cm^2^/pulse exhibited a band at 677 nm, which corresponds to an energy of 1.83 eV. The photoluminescence of the Bi_x_S_y_ film produced at 12.74 J/cm^2^/pulse demonstrated a band at 712 nm that corresponded to an energy of 1.74 eV. An emission PL peak centered at 729 nm, corresponding to an energy of 1.7 eV as observed for porous Bi_x_S_y_ film fabricated at 15.92 J/cm^2^/pulse, which can be indexed to band-band recombination of free electrons. As the laser fluence increases, the emission peak shifts to longer wavelengths (red shift) due to the increase in particle size of Bi_x_S_y_. The sample synthesized at 6.37 J/cm²/pulse shows a broad PL emission peak centered at 672 nm (1.85 eV). This emission is due to radiative recombination of electron-hole pairs via extrinsic impurity or defect states within the bandgap, rather than quantum confinement effects. This conclusion is supported by the sample’s relatively large crystallite size, which exceeds the quantum confinement regime. Additionally, similar PL features in the range of 660–680 nm have been reported in the literature^11,37^ and are likewise attributed to defect- or impurity-related transitions in Bi_x_S_y_ films.

The porous Bi_x_S_y_ nanostructure films were found to have negative Hall coefficient, indicating their n-type conductivity type, which is consistent with the data that was published^31^. This may due to low sulfur content in the film formed during the laser ablation. As demonstrated in Table 3, the the Bi_x_S_y_ nanostructure film’s electrical conductivity and mobility decrease as the fluence increases due to the high concentration of trapping sites, formation of high density of grain boundaries, and surface states. The hopping processes that take place in Bi_x_S_y_ with the aid of charge transfer rely on these partially filled gap states and are enhanced by assistance from free holes. Furthermore, the conductivity of the Bi_x_S_y_ films is found to decrease with a rise in the laser fluences due to the evaporation of sulfur atoms, altering stoichiometry and creating a less conductive phase.

**Table 3:** Influence of laser fluences on mobility and electrical conductivity of Bi_x_S_y_ films.

Laser fluence (J/cm^2^/pulse)	Conductivity (Ω·cm)^−1^ × 10^− 2^	Mobility (cm^2^/V.s)
6.37	3.78	2.91
9.55	2.95	2.37
12.74	1.08	1.72
15.92	0.30	0.58

**Fig. 11:**
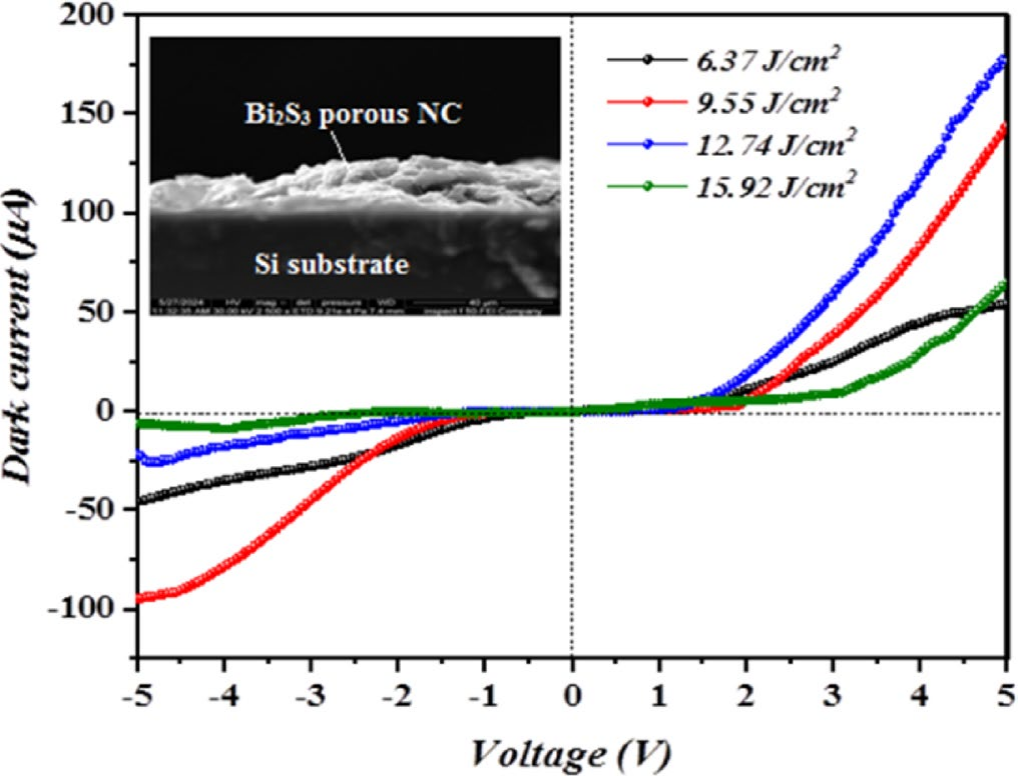
Dark I–V properties of isotype n- Bi_x_S_y_/p-Si HJs in forward and reverse bias synthesized at various laser fluences. Inset is the cross section FESEM image of the heterojunction prepared at 12.74 J/cm^2^.

Figure 11 illustrates the unilluminated I-V curves of n- Bi_x_S_y_/p-Si HJs formed with various laser fluences obtained at room temperature with biased voltage ranged within − 5 and + 5 V, inset into the figure presents ~ 200 nm film thickness. These heterojunction’s current transport mechanism is controlled by the double-Schottky-diode model. In this type of heterojunction, the current transport is mainly governed by two back-to-back Schottky barriers at the semiconductor interfaces. Under dark conditions, carriers flow mainly by means of thermionic emission over the barriers, with possible contributions from tunneling in nanosized or highly doped regions, whereas the recombination of the charge carriers in the depletion regions can reduce the ideality factor.

**Fig. 12:**
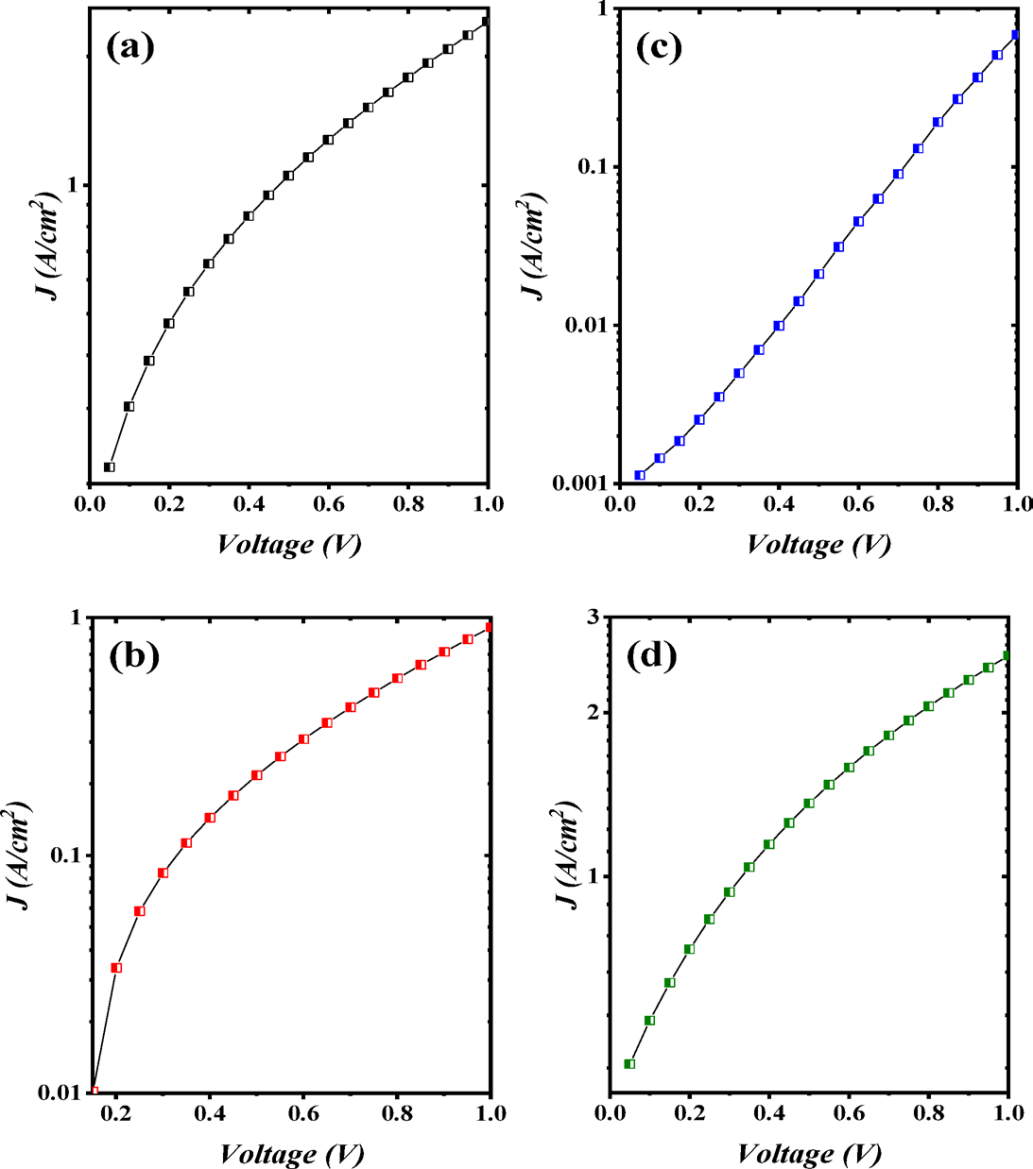
Semi-logarithmic I_f_–V plot of n- Bi_x_S_y_/p-Si HJs at various laser fluences: **(a)** 6.37, **(b)** 9.55, **(c)** 12.74, and **(d)** 15.92 J/cm^2^/pulses.

The interface state functions as a donor within the two depletion zones generating a negative space charge. As a result of the diffusion current’s dominance, the forward current rose slightly with bias up to 2 V before increasing exponentially. The reveres current was observed to rise with voltage as a result of increment in the surface leakage that allowed current to flow across the heterojunction’s edges^38,39^. Actually, the I-V properties of isotype Bi_x_S_y_/Si are regarded as a pair of back-to-back Schottky diodes. The saturation current density I_s_ of the Bi_x_S_y_/p-Si HJs prepared at laser fluence 6.37, 9.55, 12.74, and 15.92 J/cm^2^/pulse was which are estimated from semi-logarithmic relation of forward current against bias voltage (I_f_ -V) plots and the results, as shown in Fig. 12 as 0.27, 0.02, 0.001, and 0.45 µA/cm^2^, respectively.

**Fig. 13:**
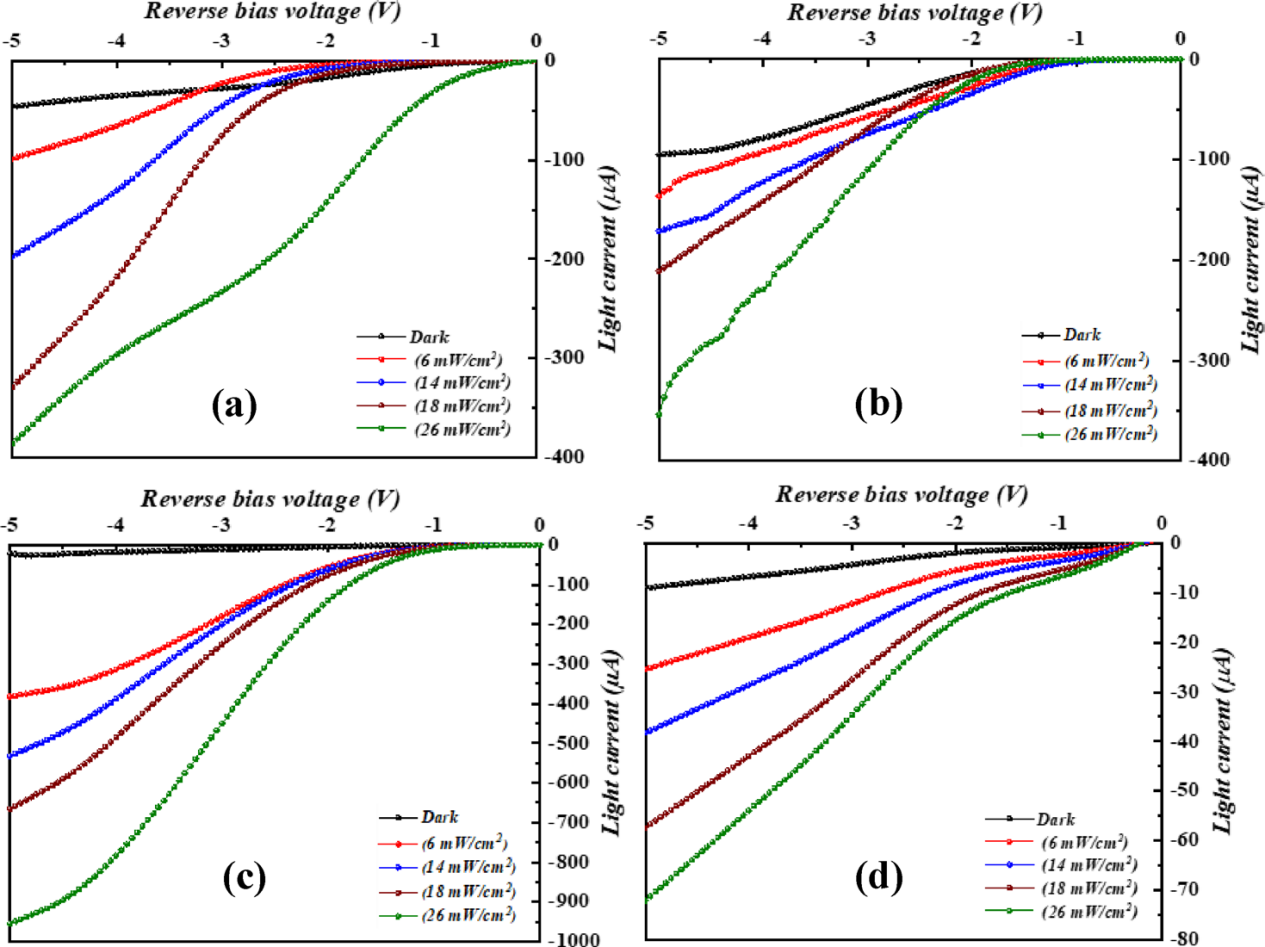
Dark and light I–V properties of n- Bi_x_S_y_/p-Si HJs PDs at various white light intensities prepared with various laser fluences: **(a)** 6.37, **(b)** 9.55, **(c)** 12.74, and **(d)** 15.92 J/cm^2^/pulse.

The ideality factor (n) of the Bi_x_S_y_/p-Si HJs was determined utilizing the following diode equation:

6 $$\:n=\:\frac{q}{{k}_{B}T}\:\left(\frac{{\Delta\:}V}{ln\frac{{\Delta\:}{I}_{f}}{{I}_{s}}}\right)$$

where k_B_ represents Boltzmann constant, q denotes the electron charge, while T is temperature of operation, I_f_ is the forward current and I_s_ represents the saturation current. By utilizing Eq. (6), the ideality factor n was evaluated and found to be 5.1, 4.6, 4.2, and 5.6, for heterojunctions prepared with 6.37, 9.55, 12.74, and 15.92 J/cm^2^/pulse, respectively, suggesting that the heterojunction produced at 12.74 J/cm^2^/pulse gives the best junction properties. Large ideality factor (n≫1) suggests that the Bi_x_S_y_/Si has poor junction properties which comes from structural defects, non-homogeneities, surface traps, interface charge, and high series resistance^40,41^. The influence of laser fluences on the photo I-V properties of Bi_x_S_y_/Si HJs PDs at various light intensities is illustrated in Fig. 13. When the light incident on the photodetector (Bi_x_S_y_ side), the e-h pair generated in the depletion region and the presence of internal and external electric fields prevent recombination of photogenerated carriers. The highest photocurrent as found for the photodetector fabricated at 12.74 J/cm^2^. As the light intensity increases, the photocurrent increases too due to the increase the number of the generated e-h pairs. The absence of saturation in the photocurrent at high light intensity, indicating that the photodetectors have linearity properties. Additionally, a continued increment in the attained photocurrent suggests a trap-assisted photoconductive gain. In detail, the occurred trap states allow prolonged carrier lifetime which in turn boost photocurrent collection phenomenon. The linear dynamic region (LDR), which indicates the linearity of Bi_x_S_y_/Si devices, was obtained from a photocurrent plot against the incident light density and determined to be 4.505, 5.095, 6.981, and 1.536 for devices produced using 6.37, 9.55, 12.74, and 15.92 J/cm^2^/pulse, respectively. On the other hand, as clarified in Fig. 13, a rise in photocurrent along increasing bias results from the enlargement of depletion region. As demonstrated, as light intensity rises, the photodetector’s photocurrent rises as well, producing a large number of e–h pairs^42,43^. The photocurrent of Bi_x_S_y_/Si HJ produced at 12.74 J/cm^2^/pulse was greater than that of the other three HJs due to the large carrier mobility, which in turns increase the diffusion length and then increasing the photocurrent. The diffusion length can be given by:

7 $$\:{L}_{d}=\:\sqrt{\frac{{K}_{B}T}{q}\mu\:\:\tau\:}\:$$

where K_B_ denotes Boltzmann constant, L_d_ represents the diffusion length of electron, q is the electron charge, is the minority carriers, and µ is the mobility.

The $\:{R}_{\lambda\:}$ is well-defined as the ratio of the photocurrent I_ph_ to the incident light power P_λ_ at a specific wavelength, as demonstrated in the formula below:

8 $$\:{R}_{\lambda\:}=\:\frac{{I}_{ph}}{{P}_{\lambda\:}}\:$$

**Fig. 14:**
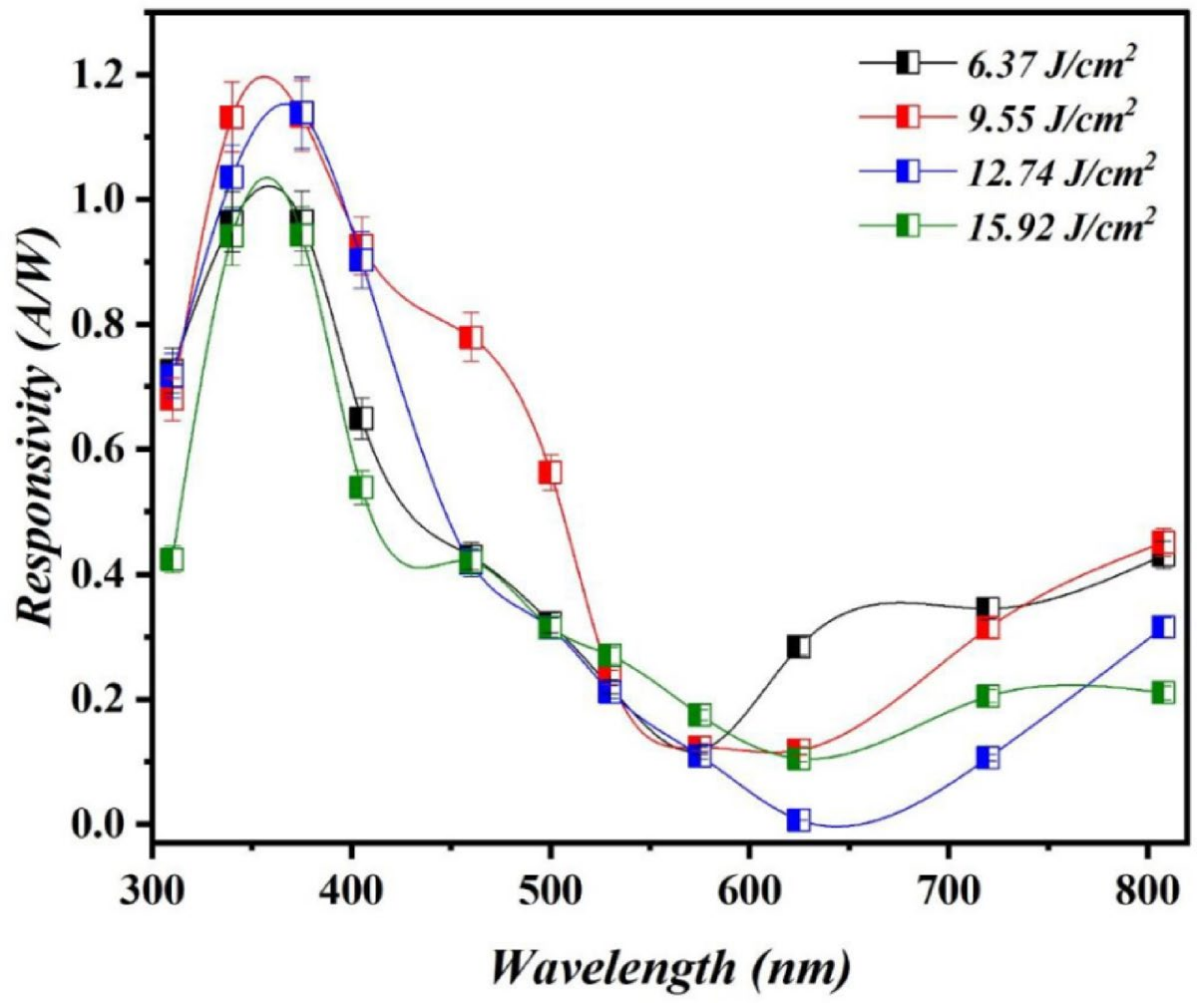
Responsivity plot of Bi_x_S_y_/p-Si photodetectors fabricated at various laser.

Figure 14 displays the spectral responsivity of Bi_x_S_y_/p-Si HJs photodetectors synthesized at various laser fluences at a bias voltage of −5 V. It is evident that all the fabricated photodetectors exhibit two response peaks. The Bi_x_S_y_/p-Si photodetector synthesized with 6.37 J/cm^2^/pulse shows two peaks of response, the first peak located at 375 nm (UV region) with a responsivity of 0.965 A/W, and the second peak found at 808 nm (NIR region) with a responsivity of 0.431 A/W.

The first peak is due to the absorption of the light at depletion region near the Bi_x_S_y_ NSs due to the high resistivity of the film, the depletion region extends toward the film more that Si substrate. Whereas the second peak is most likely results from the silicon substrate’s absorption edge^44^. The photodetector produced at 9.55 J/cm^2^/pulse has two response peaks situated at 375 nm with responsivity of 1.133 A/W, and at 808 nm with responsivity of 0.451 A/W, the photodetector fabricated at 12.74 J/cm^2^/pulse has responsivity of 1.139 A/W at 375 nm and 0.315 A/W at 808 nm, and the photodetector obtained at 15.92 J/cm^2^/pulse has responsivity of 0.942 A/W at 375 nm and 0.276 A/W at 808 nm. However, as the laser fluence increases from 12.74 to 115.92 J/cm^2^/pulse, the responsivity begins to decreases as a result of particles agglomerate of the Bi_x_S_y_, also raising the electrical resistivity of the Bi_x_S_y_ NPs^45^. The position of peak response at 375 nm can be attributed to the fact that the energy gap derived from absorbance fitting represents the optical transition threshold; however, the presence of tailing states, surface defects, or quantum confinement in nanostructured Bi_x_S_y_ can extend absorption into the visible and UV regions, resulting in increased responsivity at shorter wavelengths. High-energy UV photons generate carriers with excess energy, contributing more effectively to photocurrent, particularly in thin or defect-rich films. The responsivity of the Bi_x_S_y_/p-Si photodetectors at 808 nm was less than that at 375 nm due to the widening of the depletion region towards the Bi_x_S_y_ side film which means the presence of high electric field on the film’s surface that prevents the recombination of photogenerated carriers.

**Fig. 15:**
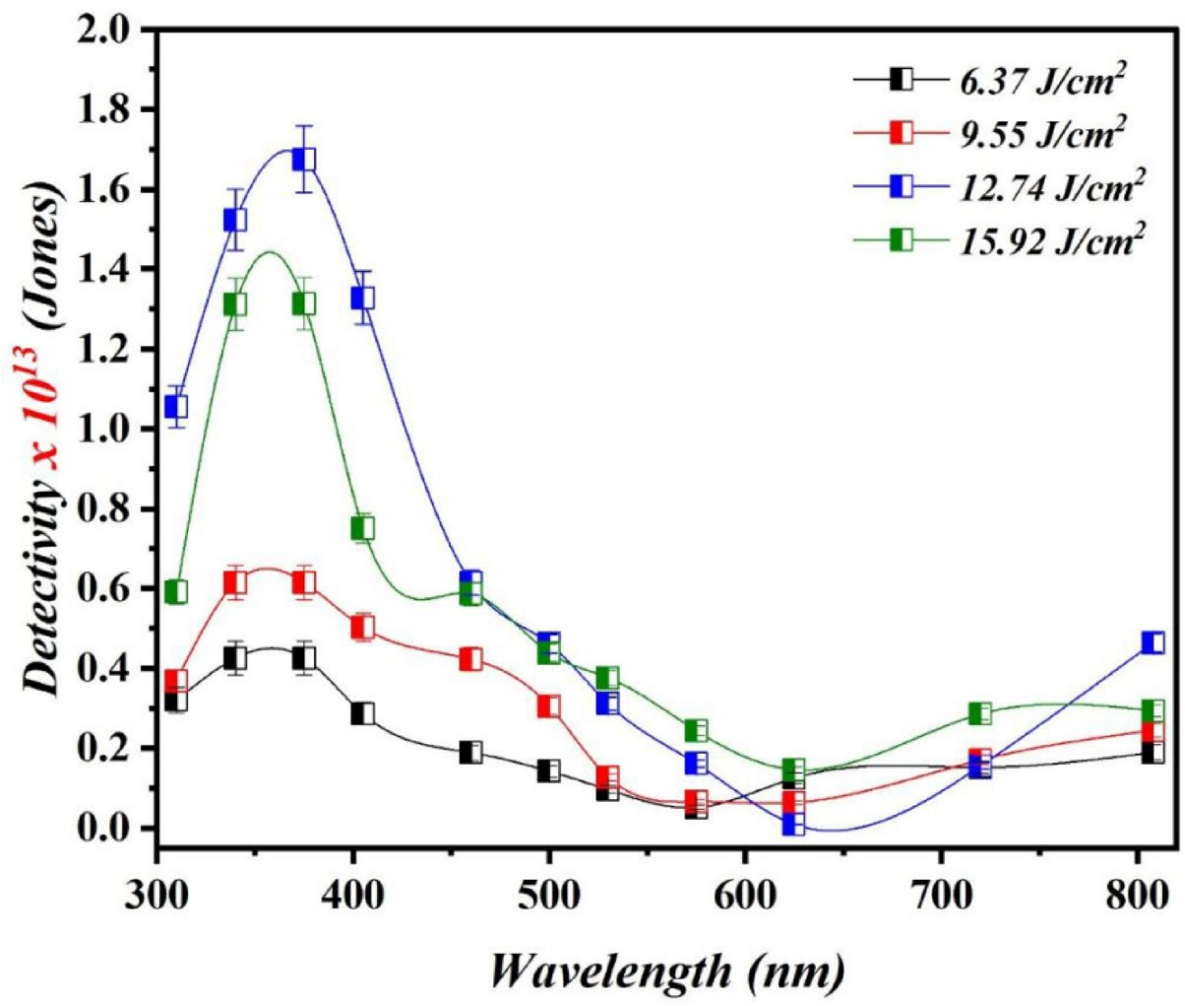
D* of Bi_x_S_y_/p-Si PDs fabricated at various laser fluences.

Figure 15 depicts the specific detectivity (D*) of Bi_x_S_y_/p-Si HJs PDs, which was calculated from the following relationship:

9 $$\:\mathrm{D}\mathrm{*}{\uplambda\:}\:=\frac{{R}_{\lambda\:}{\left(A\right)}^{\frac{1}{2}}}{\sqrt{2q{I}_{d}}\:}\:$$

where A is the photodetector sensitive area and I_d_ is the dark current of the photodetector. The photodetector prepared with 12.74 J/cm^2^/pulse has a detectivity of 1.68 × 10^13^ Jones at 375 nm, which is greater than that of the photodetectors obtained with other laser fluences. The justification for this result is the increasing the laser fluence causes the noise current in the photodetector to increase, hence decreasing detectivity, since the detectivity strongly depended on noise current and responsivity^46,47^. Table 4 displays the photodetector’s figures of merit at a bias voltage of −5 V as a function of the laser fluence. From detectivity, the noise equivalent power, or NEP, was computed. A lower NEP indicates improved weak signal detection capability of the photodetector.

**Fig. 16:**
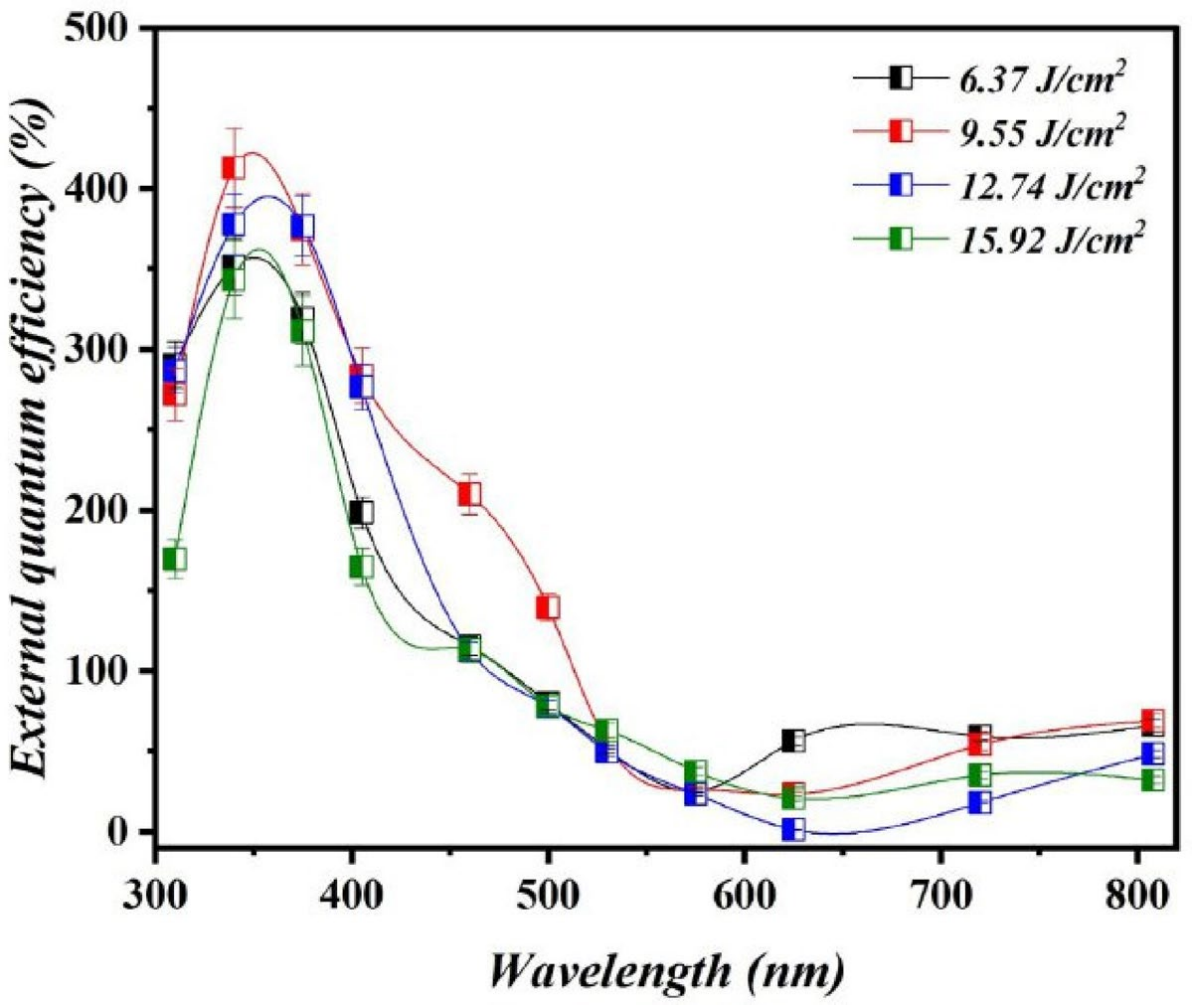
External quantum efficiency (EQE) of Bi_x_S_y_/p-Si PDs fabricated at various laser fluences operated at bias voltage of −5 V.

The external quantum efficiency ($\:\eta\:$) of photodetectors fabricated at various laser fluences is shown in Fig. 16. The external quantum efficiency can be defined as the ratio of the number of photogenerated electrons to the number of incident photons as shown in the following equation:

10 $$\:\eta\:=\frac{1240\:{R}_{\lambda\:}}{\lambda\:\left(nm\right)}$$

The maximal $\:\eta\:$ (%) was 376.52% at 375 nm for the photodetector produced with 12.74 J/cm^2^/pulse. The $\:\eta\:$ was greater than unity owing to the broadening of the depletion zone width brought about by an increase in the reverse bias voltage. Under high reverse bias voltage, the depletion region at the Bi_x_S_y_/Si interface broadened, causing in an external quantum efficiency (EQE) that exceeded unity. This expansion improves the built-in electric field, allowing for more efficient separation and collection of photogenerated carriers. In this context, the term “completely depleted” refers to the full extension of the depletion region across the active junction area where light absorption and carrier generation occur, rather than the depletion of the entire Bi_x_S_y_ layer. This condition reduces carrier recombination and increases photocarrier collection, resulting in photoconductive gain and higher EQE values^48–50^. Since the particle agglomeration negatively affects the performance of the device^51^.

**Table 4:** Figures of merit of the photodetectors fabricated with various laser fluences at 375 nm.

Laser fluence (J/cm^2^)	$\:{\mathbf{R}}_{\boldsymbol{\uplambda\:}}$ (A/W)	D*_λ_ (Jones) ×10^13^	NEP (pW)	$\:\boldsymbol{\upeta\:}$ (%)	Ideality factor (*n*)	I_s_ (µA/cm^2^)
6.37	0.965 ± 0.016	0.426 ± 0.013	0.235 ± 0.051	319.19 ± 5.45	5.1	0.27
9.55	1.133 ± 0.042	0.615 ± 0.015	0.163 ± 0.050	374.64 ± 6.37	4.6	0.02
12.74	1.139 ± 0.048	1.680 ± 0.026	0.060 ± 0.011	376.52 ± 1.33	4.2	0.001
15.92	0.942 ± 0.019	1.310 ± 0.021	0.076 ± 0.013	311.47 ± 4.24	5.6	0.45

**Fig. 17:**
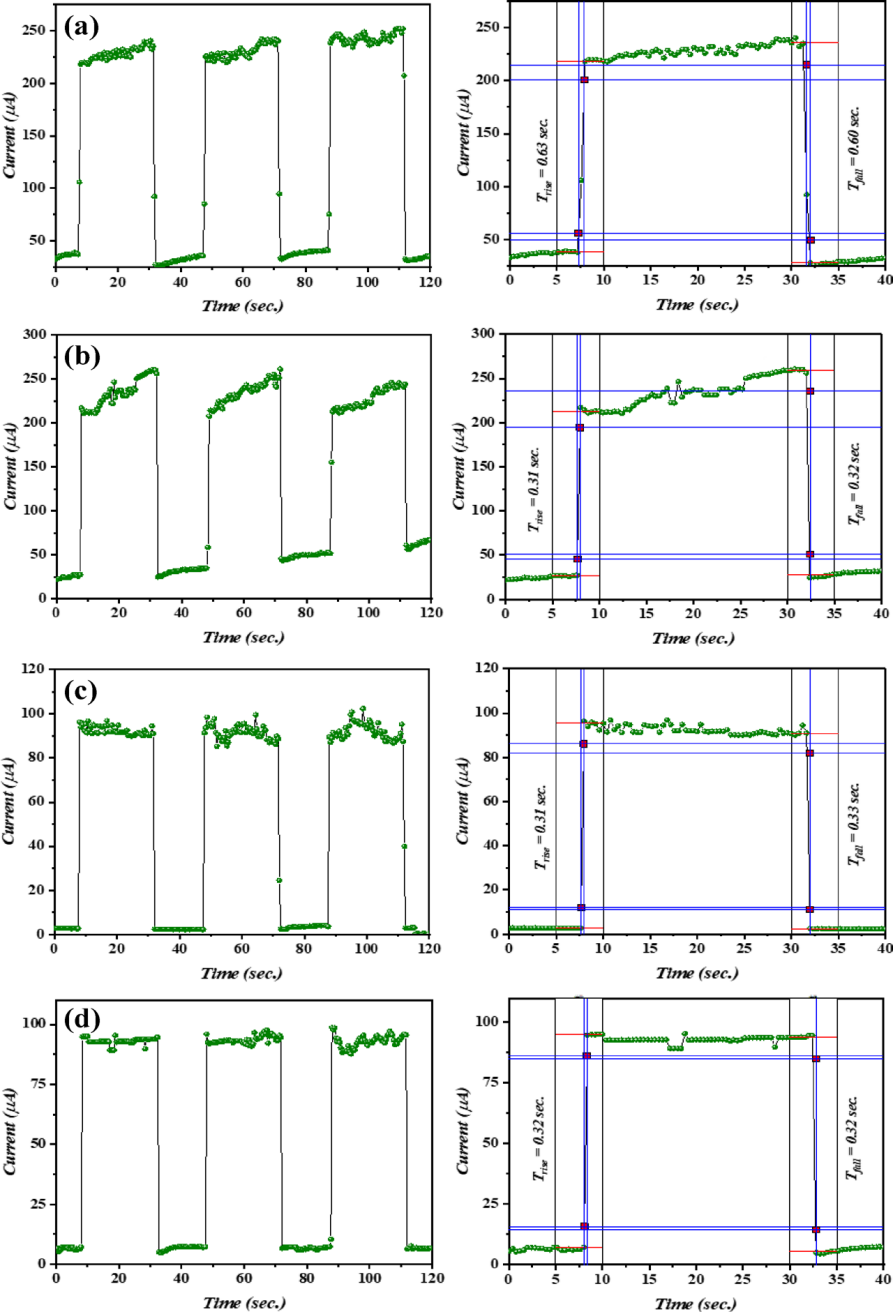
I–t curves of Bi_x_S_y_/p-Si PDs fabricated at various laser fluences: **(a)** 6.37, **(b)** 9.55, **(c)** 12.74, and **(d)** 15.92 J/cm^2^/pulse with response and recovery times.

As depicted in Fig. 17, an ON/OFF measurement was carried out to examine the response/recovery time and switch behavior of the manufactured photodetectors at various laser fluences. The measurements herein were determined over a 20 s duration with a light intensity of 26 mW/cm^2^ with different ON/OFF ratios.

The highest value of ON/OFF was 23.75 for the photodetector prepared at 12.74 J/cm^2^, as shown in Table 5. According to Fig. 17, the resulting current often rises rapidly to a certain value as laser fluences are increased. It was found that the photo-responsive behavior co-dependently upon the laser fluences utilized. For heterojunctions manufactured with 6.37 J/cm^2^/pulse, the photocurrent worth of the photodetector rapidly grew to its maximal at 0.31 s response time and then dropped to its initially value at 0.32 s recovery time. The photodetector fabricated with 9.55 J/cm^2^/pulse clarifies that its photocurrent maximal increased at a response time of 0.63 s and gradually decreased to its begin value (dark current) with a decay time of 0.60 s. The photocurrent of the photodetector, which was constructed with 12.74 J/cm^2^/pulse, peaked at a response time of 0.31 s and then decreased to its lowest value (I_d_) with a recovery time of 0.33 s. Whereas the photodetector was fabricated with 15.92 J/cm^2^/pulse, its photocurrent obtained at the maximal response time of 0.32 s and declined to its minimum value at a decay time of 0.32 s, as depicted in Fig. 17. The present findings clarifies that the Bi_x_S_y_/p-Si PDs designed at 15.92 J/cm^2^/pulse has excellent switching traits.

**Table 5:** Rise, fall time, and ON/OFF ratio of the photodetectors.

Laser fluence (J/cm^2^)	t_rise_/t_fall_	ON/OFF
6.37	0.63/0.60	9.48
9.55	0.31/0.32	10.4
12.74	0.31/0.33	23.75
15.92	0.32/0.32	11.75

**Fig. 18:**
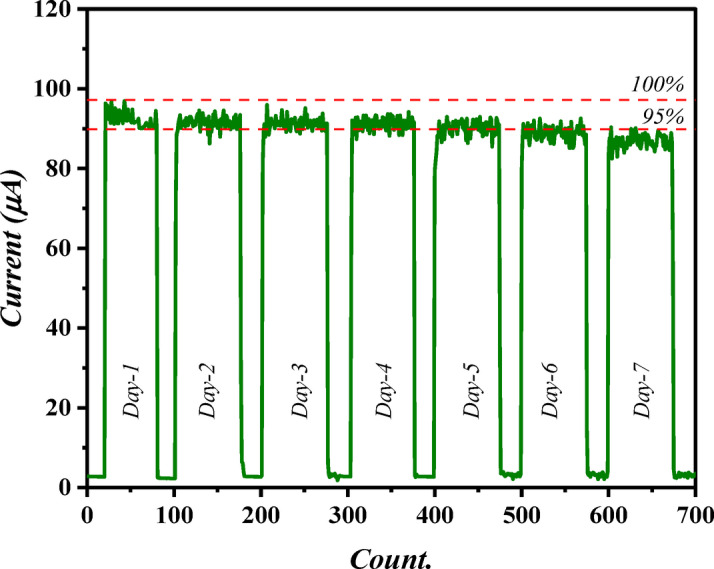
Long-term stability test of photodetector fabricated using laser fluence of 12.74 J/cm^2^.

The long-term stability test for photodetector attained using laser fluence of 12.74 J/cm^2^ is demonstrated in Fig. 18. Interestingly, the demonstrated device suggests a stable behavior over 7 multiple days as presented in the figure. However, a slight alteration in the photocurrent value was noticed over 5 days where the fabricated photodetector degraded with about 5% of its initial photocurrent value.

Table 6 presents comparison of the demonstrated work as compared to other reported data in the literature.

**Table 6:** Comparison of the attained figures-of-merits to other reported literature.

Sample	$\:{\boldsymbol{R}}_{\boldsymbol{\lambda\:}}$ (A/W)	$\:{\boldsymbol{D}}^{\boldsymbol{*}}$(Jones)	EQE (%)	$\:{\boldsymbol{T}}_{\boldsymbol{r}\boldsymbol{i}\boldsymbol{s}\boldsymbol{e}}/{\boldsymbol{T}}_{\boldsymbol{f}\boldsymbol{a}\boldsymbol{l}\boldsymbol{l}}$ (sec.)	Ref.
Bi_2_S_3_:Nd	1.36 × 10^− 1^	3.05 × 10^10^	43	0.1/0.2	^24^
LA-Bi_2_S_3_	0.036	1.69 × 10^12^	-	-	^25^
Bi_2_S_3_	0.16	9.75 × 10^9^	50.8	0.3/0.4	^26^
Bi_x_S_y_	1.139	1.31 × 10^13^	311.47	0.31/0.33	This study

## Conclusion

For the first time, porous Bi_x_S_y_ nanostructures were synthesized by laser ablation of a boron target in a liquid environment without the use of a catalyst. The impact of laser fluence on their structural, morphological, chemical, optical, and electrical properties was systematically examined. XRD analysis showed that the obtained Bi_x_S_y_ nanoparticles were polycrystalline with an orthorhombic phase, and their crystallinity increased gradually as the laser fluence was raised. UV–Vis spectroscopy indicated a slight enhancement in absorption with fluence, accompanied by a decrease in the direct bandgap of Bi_2_S_3_ from 1.8 to 1.69 eV. PL measurements for films produced at 6.37, 9.55, 12.74, and 15.92 J/cm^2^/pulse revealed emission peaks at 672, 677, 712, and 729 nm, respectively, consistent with the UV–Vis results. Raman spectra displayed three vibrational modes whose intensities increased with fluence.

The morphological analysis showed the formation of a 3D porous grid-like structure, while the topographical characteristics displayed a uniform arrangement, resulting in an average grain size of 167 nm at 12.74 J/cm^2^/pulse. Additionally, performance characteristics were evaluated for Bi_x_S_y_/p-Si heterojunction (HJ) photodetectors fabricated at various laser fluences.

The photodetectors operated across a spectral range of 300–850 nm. The device fabricated at 12.74 J/cm²/pulse showed the highest performance among the samples, with responsivity, detectivity, and external quantum efficiency measured at approximately 1.139 A/W, 1.68 × 10^13^ Jones, and 376.52% at 375 nm, respectively. Overall, this work provides a simple and cost-effective approach for developing photodetectors that function in the visible and near-infrared regions and are suitable for detecting low-power optical signals.

The rise/fall time of the Bi_x_S_y_ p-Si nanostructure fabricated at 9.55 J/cm^2^/pulse was 0.63 s/0.60 s. The laser fluence of 9.55 J/cm^2^/pulse was strongly correlated with the performance characteristics of the Bi_2_S_3_/p-Si nanostructure.

## Data Availability

All data generated or analyzed during this study are included in this published article.
